# Unusual Suspects: Detection Probability Covaries With Vegetation Productivity and Rainfall for Camera Survey of African Leopards ( *Panthera pardus pardus* )

**DOI:** 10.1002/ece3.71346

**Published:** 2025-05-14

**Authors:** Beatrice Chataigner, Nicholas W. Pilfold, Laiyon Lenguya, Aurélien G. Besnard, Olivier Gimenez

**Affiliations:** ^1^ CEFE, Univ Montpellier, CNRS EPHE‐PSL University, IRD Montpellier France; ^2^ San Diego Zoo Wildlife Alliance San Diego California USA; ^3^ Loisaba Conservancy Nanyuki Kenya; ^4^ CEFE, Univ Montpellier, CNRS, EPHE, IRD Montpellier France

**Keywords:** African leopard, camera trapping, causal inference, detection probability, occupancy, *Panthera pardus*

## Abstract

Maximizing detection probability of elusive species enhances the robustness of population and occupancy estimates, which are essential for supporting impactful conservation strategies. Although the number of camera trap studies on leopards is increasing, few have assessed the drivers influencing leopard detection specifically. We used occupancy modeling embedded in a causal inference framework to provide four biologically robust site use models against which to test the influence of six factors likely influencing leopard detectability at the level of encounter probability, trigger probability, and image quality. In this study, vegetation productivity moderated by rainfall was the top predictor of leopard detectability associated with three of the four site use models. While optimizing detection probability improves estimates of population parameters, the cost‐effectiveness of the study designs is also an essential criterion to consider for long‐term monitoring of elusive species. Camera trap placement involves minimal cost in the early stages of the grid design. Our results suggest that setting cameras in microhabitats of moderate productivity improved leopard detectability in the wet season. This study can inform the design of camera trap studies occurring in semi‐arid bushland ecosystems to improve estimates of leopard population and occupancy.

## Graphical abstract

Camera trapping studies are increasingly used to derive population and occupancy estimates of leopards; however, little is known about the drivers influencing leopard detection when using such devices. We used occupancy modeling embedded in a causal inference framework to identify factors influencing leopard detectability. Our results suggest that setting cameras in microhabitats of moderate productivity improved leopard detectability in the wet season.

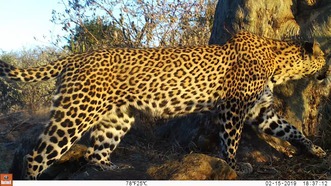

## 1 Introduction

Apex predators play an important role in maintaining biodiversity and ecosystem function (Ripple et al. 2014), and the depletion of their populations can have wide‐ranging ecosystem consequences (Estes et al. 2011; Ripple et al. 2016; Van Cleave et al. 2018). Due to the high metabolic demands driven by endothermy and body size, large carnivores require access to abundant prey and expansive habitats (Cardillo et al. 2004; Ripple et al. 2016). These food requirements and wide‐ranging behavior often bring them into conflict with humans and their livestock (Cardillo et al. 2004; Ripple et al. 2016), where they face a direct mortality risk from persecution and retaliation (Woodroffe and Frank 2005; Kissui 2008). In addition to poaching, other indirect threats, such as habitat loss and degradation, utilization, and depletion of prey, have hastened declines in large carnivore populations and geographic ranges, rendering them vulnerable to extinction (Ripple et al. 2016).

Because they occur at low densities over large areas (Cardillo et al. 2004), large carnivores have low detection probabilities, which render their monitoring even more challenging. If not accounted for, imperfect detection of a species (“false absence”) leads to underestimated abundance estimates (Mackenzie 2005). The methods used to estimate detection probabilities of individual animals require sufficient sample sizes (Mackenzie et al. 2002). Yet, territorial species occurring at low densities such as large carnivores typically translate into small sample sizes that lead to imprecise population estimates (MacKenzie et al. 2005). Increasing sampling effort can overcome this issue, but it is expensive. Another approach consists of considering a state variable requiring smaller sampling effort, such as the proportion of area occupied by a species (i.e., occupancy) (MacKenzie et al. 2005). Occupancy models (Mackenzie et al. 2002) are hierarchical models that explicitly account for detection probabilities; therefore, allowing distinguishing between true absences of animals and animals that were simply not detected although present at the site. Maximizing detection probability enhances the robustness of occupancy estimates of elusive species (Kellner and Swihart 2014).

**FIGURE 1:**
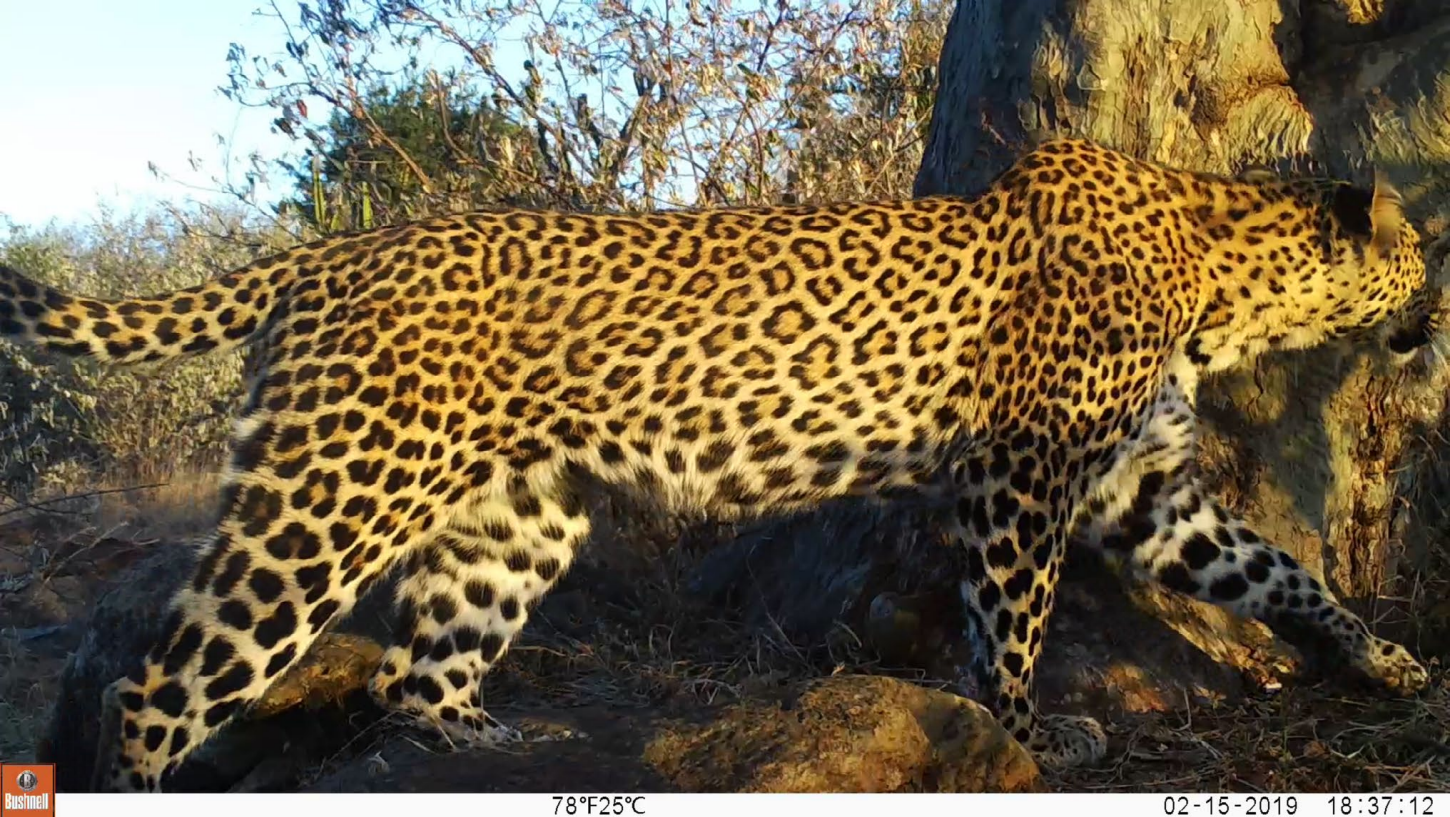
Image of leopard detected with a camera trap from an array designed for leopard density estimation in Loisaba Conservancy in Laikipia County, Kenya. Photo credit: San Diego Zoo Wildlife Alliance.

Camera trapping is a widely used non invasive technique to collect presence/absence data to infer occupancy estimates of elusive species (Rovero et al. 2013; Burton et al. 2015). Camera traps are passive detectors with automatic cameras attached to passive infrared (PIR) sensors that trigger the capture of an image when warm‐bodied animals pass through the sensor beams (Rovero et al. 2013) (Figure 1), and numerous factors can affect their function as a survey tool. The probability of an animal being detected by a camera trap is conditional on both the probability of the animal being present at the site (i.e., site use probability) and on the probability of being photographed if present (i.e., detection probability). The detection probability of camera traps can be hierarchically broken down into three conditional probabilities (Hofmeester et al. 2019). First, the probability of the target species crossing the detection zone in front of the trap (i.e., encounter probability); second, the probability of triggering the camera when entering the detection zone (i.e., trigger probability); third, the probability of the camera taking an image of good enough quality to identify the species or individual (i.e., image quality). Each of these conditional probabilities can be affected by three types of factors: the biological traits of the target species (i.e., the environmental characteristics, as well as the camera trap grid design [Hofmeester et al. 2019]). Among the species biological traits, animal detection can be affected by body size (Hofmeester et al. 2017) and behavior (e.g., speed of movement [Findlay et al. 2020], trap shyness [Wegge et al. 2004]). Environmental and ecological drivers for leopards may include: (i) bioclimatic conditions such as relative humidity (Meek et al. 2014; Hofmeester et al. 2019) and temperature (Cho et al. 2012; Welbourne et al. 2016; Hofmeester et al. 2017); (ii) habitat features such as vegetation density at the site, large trees (Smith 1977; Bothma and Coertze 2004; Stein et al. 2015; Balme et al. 2017) and trails. Additionally, many grid design characteristics are known to influence detectability. These include the survey effort (Mackenzie and Royle 2005), the number of cameras per site (Stokeld et al. 2015; Hofmeester et al. 2021; Kolowski et al. 2021), the frequency of camera maintenance (Hofmeester et al. 2019), the camera type (Rovero et al. 2013), the camera placement nearby habitat features channeling animal movement (Hofmeester et al. 2019), the presence of bait or lure (Du Preez et al. 2014), the camera height and orientation (McIntyre et al. 2020), and the camera setup (Findlay et al. 2020). Recording the variation in these factors to be considered as covariates in camera trap studies is vital, because they influence the probability of detecting the species, which in turn affects the parameter estimates (occupancy) drawn from the analysis of camera trap data (Mackenzie 2005). Camera trap sampling designs therefore must be rigorously tested to optimize the detection of the target species (Hofmeester et al. 2019).

African leopards ( *Panthera pardus pardus* ) are considered Vulnerable to extinction by the International Union for the Conservation of Nature (Stein et al. 2025). African leopard populations suffer from habitat loss and prey depletion (Rosenblatt et al. 2016; Loveridge et al. 2022), poorly managed trophy hunting (Balme et al. 2012; Loveridge et al. 2022) and poaching for body parts and skins (Stein et al. 2025). Despite the severity of the threats and ongoing range contraction, studies addressing distribution and population size to inform conservation efforts remain limited in comparison to those focused on behavioral aspects (Balme et al. 2014; Jacobson et al. 2016). Few countries have national strategies for leopards. Kenya, in which our study area is situated, has carnivore conservation strategies for lions ( *Panthera leo* ), spotted hyenas ( *Crocuta crocuta* ), cheetahs ( *Acinonyx jubatus* ) and wild dogs ( *Lycaon pictus* ) but not for leopards.

Rigorous estimates of leopard population size and distribution are necessary to assess the effectiveness of conservation programs (Strampelli 2015). However, leopards are notoriously difficult to monitor as they are shy, solitary, and nocturnal, with wide‐ranging patterns and naturally occur at low densities (Balme et al. 2009). There is an increasing number of contemporary studies on leopard distribution relying on camera trapping (Carter et al. 2015; Miller et al. 2018; Strampelli et al. 2018), yet few have assessed the drivers influencing leopard detection specifically. In this study, we used observation data from camera traps that we processed through a causal inference framework to identify factors influencing leopard detectability following the hierarchal conditional probabilities from Hofmeester et al. (2019). We hypothesized that the variables driving success in the three components of detection probability would be different. First, we predicted that encounter probability would be positively influenced by the presence of a trail or the presence of a tree opposite the camera. Second, we predicted trigger probability to be negatively correlated with camera height, trail, and vegetation productivity especially during the rainy season, but positively associated with the presence of a tree facing the trap. Last, we expected the probability of obtaining good quality images would decrease during the rainy seasons and with higher camera height. The rationale supporting these hypotheses is reported in Table 1. Based on our results, we discuss camera trapping study design for the effective monitoring of leopards to better inform conservation strategies.

**TABLE 1:** Hypotheses tested and the expected effect of related covariates on each conditional probability of site use, encounter, trigger, or image quality.

Hypothesis	References	Covariates	Predicted effect of covariates	Additional covariates to condition on as a result of the backdoor criterion analysis	Probability affected by the covariates	Mechanism
Leopards favor habitat of high vegetation productivity providing adequate resources for prey and enough hunting cover.	Balme et al. 2007	Average vegetation productivity at site (NDVI)	NDVI (+)	Trees; river 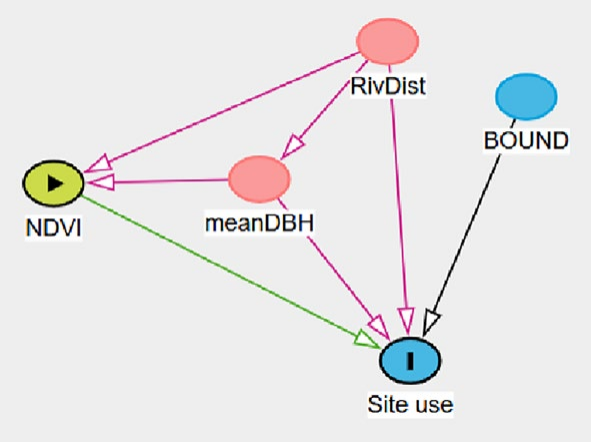	Site use probability	Contact with CT
Leopards favor riparian habitats	Abade et al. 2014; Davidson et al. 2012	Distance to nearest river (River)	River (−)	None 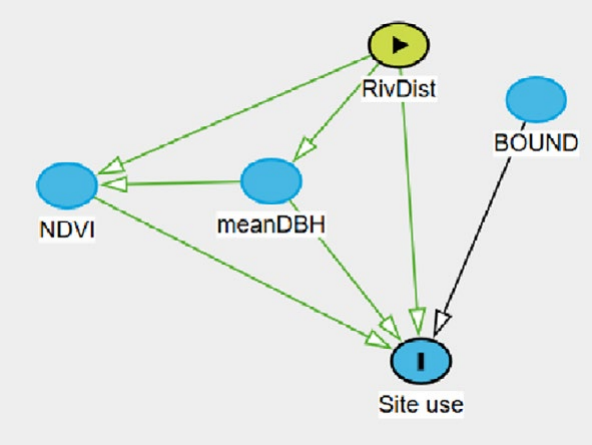	Site use probability	Contact with CT
Leopards favor sites with large trees suitable for hoisting carcasses	Smith 1977; Balme et al. 2017	Mean diameter at breast height of trees present at site (Trees)	Trees (+)	River 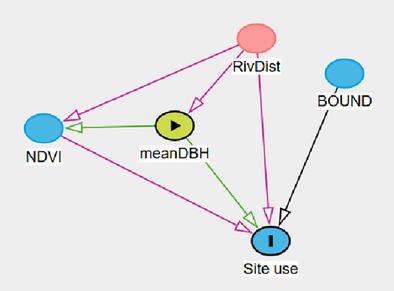	Site use probability	Contact with CT
Leopards tend to avoid human by keeping away from human settlements (using protected area boundaries as a proxy for presence of human settlements)	Balme et al. 2010; Havmøller et al. 2019	Distance to conservancy border (Boundary)	Boundary (+)	None 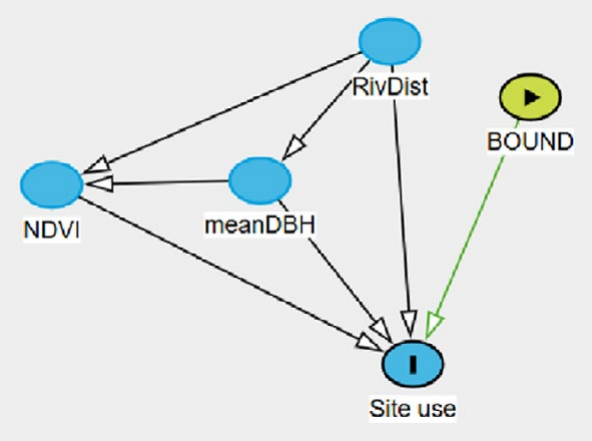	Site use probability	Contact with CT
Leopards use trails to ease movement thus having greater chances to enter the detection zone of CT facing trails	Hunter et al. 2013; Strampelli et al. 2018; Harmsen et al. 2010; Kolowski and Forrester 2017	Presence of a trail at camera site (Trail)	Trail (+)	NA	Encounter probability	Contact with CT
Leopards use large trees for hoisting prey, scent marking, and resting	Stein et al. 2015; Balme et al. 2017; Nowell and Jackson 1996; Allen et al. 2020	Diameter at breast height of tree within camera view shed (CT_Tree)	CT_Tree (+)	NA	Encounter probability	Contact with CT
Low ambient temperature drain the batteries quicker resulting in slower response time of the camera and lower PIR sensor sensitivity	Cho et al. 2012	Mean daily temperature (Temp)	Temp (+)	NA	Trigger probability	PIR sensor sensitivity
At low ambient temperature, the PIR sensor better detect a difference in heat between leopards and the environment and therefore increase the chance of triggering the camera	Welbourne et al. 2016; Hofmeester et al. 2017; McIntyre et al. 2020; Swann et al. 2004	Mean daily temperature (Temp)	Temp (−)	NA	Trigger probability	PIR sensor sensitivity
Increasing CT height increases the distance between the animal and the CT	Findlay et al. 2020; McIntyre et al. 2020	Height of camera off the ground (CT_Height)	CT_Height (−)	NA	Trigger probability	PIR sensor sensitivity
Sites with high vegetation productivity in rainy season are associated with quick vegetation growth, which triggers the CT and drains batteries quicker resulting in a slower response time of the camera and lower PIR sensor sensitivity	Hofmeester et al. 2017	Average vegetation productivity at site (NDVI) mean daily rainfall (Rain)	[NDVI × Rain] (−)	NA	Trigger probability	PIR sensor sensitivity
Sites with high vegetation productivity negatively affects trigger probability because vegetation affects transmission of infrared radiation	Hofmeester et al. 2017; Özyavuz et al. 2015	Average vegetation productivity at site (NDVI) mean daily rainfall (Rain)	NDVI (−)	NA	Trigger probability	PIR sensor sensitivity
Leopards spend more time in front of the camera if placed opposite a large tree that they use for scent marking or for hoisting prey	Smith 1977; Bothma and Le Riche 1984	Diameter at breast height of tree within camera viewshed (CT_Tree)	CT_Tree (+)	NA	Trigger probability & Image quality	Retention time in front of CT
The presence of rain on the sensor of the CT lowers the sensitivity of the passive infrared (PIR) sensor	Meek et al. 2014; Hofmeester et al. 2019	Mean daily rainfall (Rain)	Rain (−)	NA	Trigger probability	PIR sensor sensitivity
Trails ease leopard movement and thus, increase speed and directionality which in turn decreases the time spent in front of CT, leading to lower trigger probability and lower image quality	Hunter et al. 2013; Hofmeester et al. 2019; McIntyre et al. 2020; Rowcliffe et al. 2011	Presence of a trail at camera site (Trail)	Trail (−)	NA	Trigger probability & Image quality	Retention time in front of CT & animal identification
Rain blocks the field of view of the camera	Hofmeester et al. 2019	Mean daily rainfall (Rain)	Rain (−)	NA	Image quality	Animal identification
The distance between the animal and the CT increases with increasing CT height, therefore rendering animal identification more challenging	Findlay et al. 2020; McIntyre et al. 2020	Height of camera off the ground (CT_Height)	CT_Height (−)	NA	Image quality	Animal identification
Sites with high vegetation productivity in rainy season are associated with quick vegetation growth, which triggers the CT and drains batteries quicker potentially resulting in lower flash output	Hofmeester et al. 2017	Average vegetation productivity at site (NDVI) mean daily rainfall (Rain)	[NDVI × Rain] (−)	NA	Image quality	Animal identification
Low ambient temperature drains the batteries quicker resulting in lower flash output	Cho et al. 2012	Mean daily temperature (Temp)	Temp (+)	NA	Image quality	Animal identification

*Note:* Interaction effect between two covariates is symbolized by a multiplication sign “×.” Covariates are seen as continuous variables unless otherwise stated in the table. The direction of the effect is either positive (+) or negative (−) and is based on an increase in the covariates. Each conditional probability relies on one or several mechanisms inherent to camera trap (CT) functioning.

## 2 Materials and Methods

### 2.1 Study Area

**FIGURE 2:**
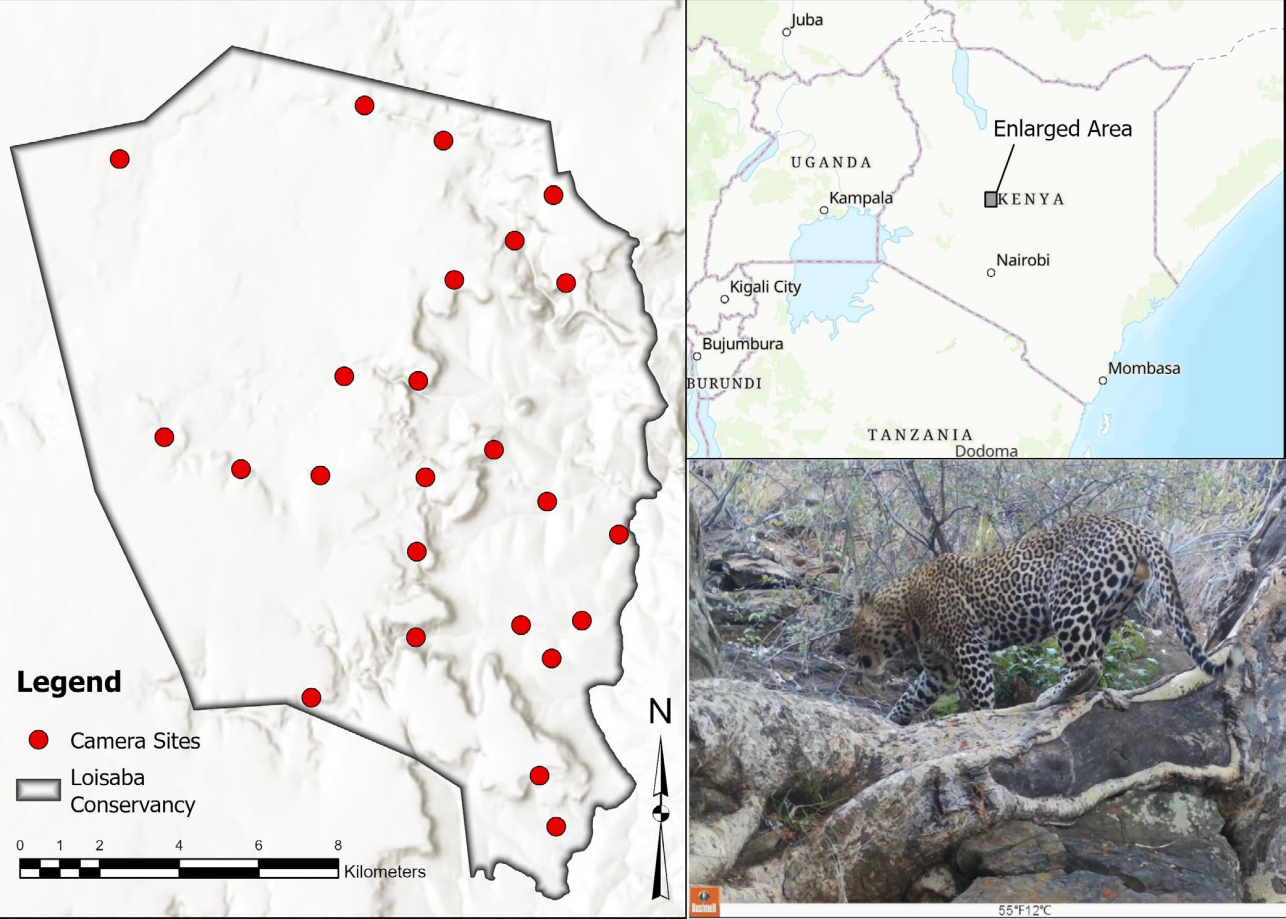
Study area image of Loisaba Conservancy in Laikipia County, Kenya. Twenty‐four cameras were deployed using 1‐day sampling occasions from February 14th to December 20th, 2019.

Loisaba Conservancy (N 0°36.41', E 36°48.23', elevation range of 1400 to 1800 m) is a 22,780 ha area managed for livestock, wildlife, and tourism located in Laikipia County in central Kenya (Figure 2). Laikipia County, an important habitat for wildlife populations within East Africa, is estimated to hold 8% of Kenya's large herbivore populations (Kinnaird and O'Brien 2012; Maggi 2014), and is one of only two regions in Kenya with substantial remaining carnivore populations (Frank et al. 2005; Georgiadis et al. 2007). This includes a population of approximately 200–250 lions (L. Frank 2011), as well as populations of leopards (Mizutani and Jewell 1998), cheetahs, spotted hyenas, striped hyenas ( *Hyaena hyaena* ), and African wild dogs ( *Lycaon pictus* ) (Frank et al. 2005). This region is also a notable exception to the national trend of declining wildlife populations (Western et al. 2009; Kinnaird and O'Brien 2012). Loisaba Conservancy is positioned within a broad matrix of private and community‐owned lands (Masiaine et al. 2020) and livestock ranching of cattle, sheep, goats, and camels is performed using traditional herding methods, employing pastoralist herders (Kinnaird and O'Brien 2012) and keeping livestock in corrals at night (L. G. Frank 1998; Ogada et al. 2003; Woodroffe et al. 2005). The northern portion of the conservancy consists of relatively flat, open grassland with scattered shrubs and acacia (*Acacia* spp.) bushes and trees. The Ewaso Narok and Ewaso Nyiro rivers meet at the southeastern edge of the conservancy (Masiaine et al. 2020). Large, rocky, and flat‐topped escarpments and drainages dominate much of the southern portions of the conservancy (Masiaine et al. 2020). The climate is semi‐arid with an average annual temperature of 24°C, and a mean annual precipitation of 603 mm (worldclim.org). Most rainfall occurs in April/May and lesser rainy seasons in July/August and October/November (Masiaine et al. 2020), but this pattern can be highly variable year to year (Barkham and Rainy 1976).

### 2.2 Data Collection

There are significant differences in the use of certain terms among the occupancy, capture recapture, and camera trap literature (Tobler et al. 2015). Here, we use the terms “site” for the location of an individual camera. We collected leopard detections with camera traps (Figure 1) set up in an array designed for leopard density estimation. The camera traps collected data through 1‐day sampling occasions from February 14th to December 20th, 2019. Twenty‐four cameras were placed to ensure that the spatial assumptions of density models were met: (i) all leopards had a non‐null chance of being detected, and (ii) the distance separating the traps ensured that each leopard territory was covered within the survey area (Green et al. 2020). The placement of cameras focused on habitats favored by leopards while avoiding sites where leopards would be unlikely to maintain territories (O'Brien and Kinnaird 2011). All cameras were placed such that one camera was available per mean home range of a female leopard, with a mean spacing of 2.3 km between each trap. Lacking information on the mean home range size of a female leopard in our study area, we considered that 30 km^2^ was a good estimate, based upon estimates of range sizes published from populations with similar densities (Parker et al. 2023). To maximize the probability of detecting leopards, camera trap stations were placed along animal trails and/or facing a suitable tree that could be used for scent marking or prey hoisting. To reduce false detections, any vegetation that might obstruct the camera's field of view was cleared. At each site, a single Bushnell 24MP Low Glow Aggressor Trail Camera (Bushnell Corporation, Kansas, USA) was set. The cameras recorded 24 h a day, using 10–15 s video capture with no delay between captures. Cameras remained on‐site permanently and were checked regularly to clear the vegetation in front of the trap, to download the memory cards, change the batteries if necessary, and ensure the set‐up remained intact. Trap maintenance was operated on a bi‐monthly basis from February to July and on a monthly basis from August to December 2019. For the analysis, we defined a “detection” as one or several leopards detected on a single day at a single site. Multiple captures of leopards at the same site on the same day were treated as a single detection.

### 2.3 Occupancy Model

To model factors influencing site use and detection probability, we derived a set of covariates from remotely sensed and in situ data collection (Table 2). We derived the Normalized Difference Vegetation Index (NDVI) using the function slope‐based vegetation index in SAGA 7.8.2. software, from the compilation and processing of available rasters of each month of 2019 from Sentinel 2 satellite, having less than 10% cloud cover (https://earthexplorer.usgs.gov/). The values of the averaged NDVI at site (NDVI) were calculated as the mean value of all pixels included in a 20 m radius buffer area around each camera trap station, located at the center of each monthly raster. In our study, sites were purposely located in dense habitats. NDVI ranged from 0.32 to 0.57, and the highest values of NDVI were correlated to sites located in closed habitats with high Terrain Ruggedness Index (TRI). Thus, in this study, sites with minimum NDVI correspond to moderate average vegetation productivity and moderate TRI when considering the whole range of habitat variation available in the study area. The distance from each camera trap station (i) to the nearest river or water point (River) and (ii) to the nearest Loisaba Conservancy boundary (Boundary) was calculated using the function *NNjoin* in QGIS (v. 10.074). We conducted field measurements to determine (i) the average diameter at breast height of trees located within a 20 m vicinity of the camera traps (Trees) and the diameter of the tree opposite to which camera traps were placed (CT_Tree), (ii) the height of camera traps off the ground (CT_Height), and (iii) whether the camera traps were facing a trail or not (Trail). We computed the average daily rainfall (Rain) and average daily temperature (Temp) of the study area by calculating the mean daily values of these variables, collated from four weather stations located around Loisaba Conservancy.

**TABLE 2:** Covariates used to model site use and detection probability of a camera survey of African leopards ( *Panthera pardus pardus* ) at Loisaba Conservancy, Kenya.

Name	Measurement	Range	Model component	Influence
NDVI	Mean Normalized Difference Vegetation Index	0.32–0.57	*λ p*	Site use, Encounter, Trigger
River	Distance to nearest river	0–1.2 km	*λ*	Site use
Trees	Mean diameter at breast height of trees within 20 m radius of camera site	0–2.4 m	*λ*	Site use
Boundary	Distance to conservancy border	0.3–6.8 km	*λ*	Site use
CT_Tree	Diameter at breast height of tree within camera viewshed	0–3.4 m	*p*	Encounter, Trigger, Image
Trail	Presence of a trail at camera site	1/0	*p*	Encounter, Trigger, Image
Rain	Mean daily rainfall	0–57.6 mm	*p*	Encounter, Trigger, Image
Temp	Mean daily temperature	16.5°C–24.9°C	*p*	Encounter, Trigger
CT_Height	Height of camera off the ground	0.1–1.4 m	*p*	Trigger, Image

We used an occupancy framework for detections at the species level, rather than individual identification, thus providing a larger sample size of detections to model the effect of covariates on detection probability and site use of leopards (Tobler and Powell 2013). Because we used data from an existing sampling design that purposely selected sites in suitable habitat for leopard rather than a random grid, we expected occupancy estimates to be overestimated and the variability range of habitat covariates to be restricted. Thus, we focused our results interpretation on covariate discrimination only, without discussing occupancy estimates. Occupancy models use replicated detection/non‐detection surveys to estimate the probability of detecting a species (*p*) and derive unbiased probabilities of sites being used by the species (*Ψ*) (Mackenzie et al. 2002). A critical assumption of occupancy modeling is that the probability of detection is constant across all sites and surveys during the survey period (i.e., primary occasion), or is a function of time‐ or site‐specific covariates (Mackenzie and Royle 2005). Variation in local abundance, however, can create heterogeneity in a species' detection probability (Royle 2006; Mackenzie et al. 2018). Thus, exploiting the relationship between abundance, detection and occupancy (O'Connell et al. 2011), the Royle and Nichols (2003) model was developed from temporally replicated detection–non‐detection data, to estimate site‐specific heterogeneity in a species' detection probability (*p*), derived from site‐specific local abundance of the species (*λ*). We used the Royle and Nichols model as a camera trap station‐level model (Tobler et al. 2015) that can account for detection heterogeneity due to higher visit frequency of camera trap stations located close to an animal's activity center.

Leopards maintain territories with stable activity centers over years in our system, and therefore we assumed that changes in occupancy within a primary occasion of one year should be random. Thus, we were able to relax the closure assumption, so that the presence of leopard at a given site could be interpreted in terms of units that are “used,” rather than “occupied” (Mackenzie et al. 2018). However, with the closure assumption relaxed, and because we could not account for animal movement between sites and within sites, multiple encounters at sites could simply reflect consecutives visits of a single individual with more spatially concentrated space use (Broadley et al. 2019; Neilson et al. 2018). Consequently, we interpreted our estimator (*λ*) as the intensity of site use, rather than an index of local abundance at site.

### 2.4 Model Selection

We used the method described by Stewart et al. (2023) that allows for testing competing causal assumptions of the site use component in the occupancy model and selecting the set of covariates predicting best detection probability for each competing site use hypothesis. We first used a causal inference analytical framework (Structural Causal Modeling, (Pearl 2000) to create our top model for *λ* following Stewart et al. (2023)). We aimed to quantify the total causal effect of predictors on the probability that leopards will use the site, represented by *λ*. Drawing from literature, we developed a directed acyclic graph (DAG) for the causal effects of covariates influencing site use (Table 1). We assessed the DAG's assumptions against our data where continuous covariates were standardized on a *z*‐scale. Whenever DAG assumptions were not supported by our data because of ambiguous results due to our small sample of sites (Shipley 2002), the combination of variables of these assumptions were excluded from the set of candidate models. We then applied the “backdoor criterion” to the DAG for each predictor as to develop relevant models aimed at assessing the effects of each predictor on the outcome of interest (*λ*). The “backdoor criterion” is meant to determine the minimum set of covariates to condition on, to close non‐ causal paths and allow a causal interpretation of the slope of the predictor of interest, conditional on the DAG's assumptions being true, while avoiding common interpretational problems such as confounding, over control, or collider biases (McElreath 2020; Arif and MacNeil 2023).

Following the creation of our site use models, we analyzed the fit of detection covariates using Akaike information criterion for small samples (AIC_C_; Burnham and Anderson 2002), as causal inference approaches are complementary to information criterion, and AIC_C_ is robust at selecting detection covariates (Stewart et al. 2023). Continuous variables were tested for collinearity using Pearson's correlation test and not included in the same model if *r* > 0.6 (Green 1979). Correlations between continuous and categorical covariates were assessed using a Student *t*‐test, and not included in the same model if the *p*‐value of the test was < 0.05. To model detection, we varied all possible combinations of detection factors, while keeping the site use component from the causal inference static. We fitted occupancy models in the maximum likelihood framework using the R package *unmarked* (Kellner et al. 2023). Goodness of fit was assessed using the R packages *mb.gof.test* in the *AICcmodavg*, which computes the MacKenzie and Bailey (2004) goodness‐of‐fit test for single season occupancy models based on Pearson's chi‐squared and extend it to Royle and Nichols (2003) occupancy models. Covariates were deemed to explain heterogeneity in leopard site use intensity (*λ*) and detection probability (*p*) if the *β*‐coefficient ± 1.96 × SE did not include zero (MacKenzie and Bailey 2004). All statistical tests were completed using R Studio v2021.09.1 utilizing R v4.1.2 (R Core Team 2020).

## 3 Results

**FIGURE 3:**
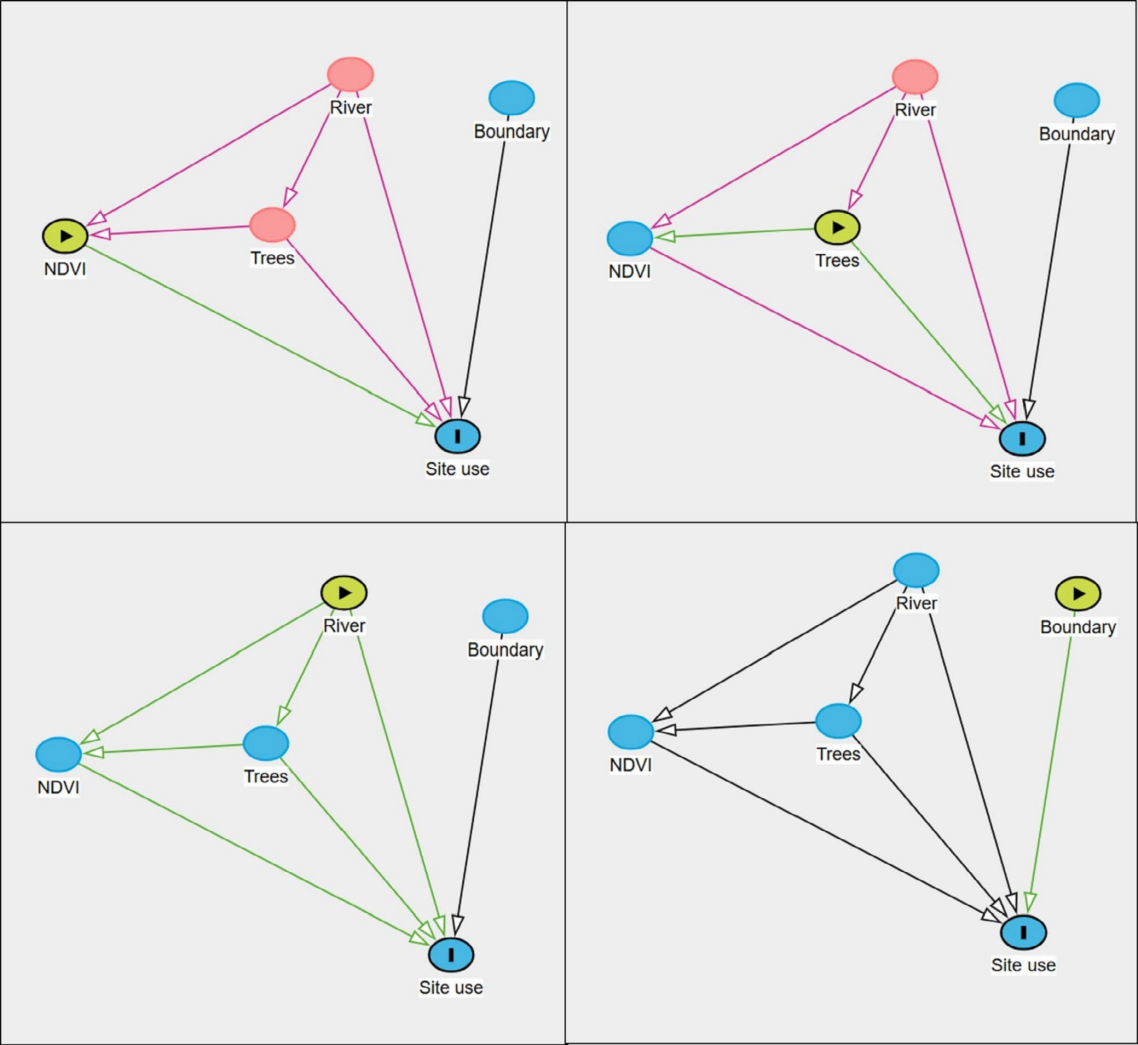
Direct acyclic diagram (DAG) displaying the causal effects of observable variables on site use probability. Observable variables include the averaged normalized difference vegetation index at site (NDVI), the distance to river (River), the distance to Loisaba boundary (Boundary), the average diameter at breast height of trees located in the vicinity of the camera traps (Trees). Light green circles indicate the variable of interest and red circles indicate the need of covariate in the model to satisfy backdoor criterion.

A survey effort of 4249 camera‐trap days total across 24 camera stations resulted in an average of 177 sampling occasions per station. Among the 164 images that contained a leopard, only 4 were not identifiable to species. Thus, we recorded a total of 160 leopard photographic events. Using the backdoor criterion analysis applied to our DAG, we identified four site use models (Figure 3). The site use model assessing the effect of “NDVI” was adjusted by adding the main effect of “Trees” and “River” (Model A). The site use models assessing the effects of “River” (Model B) and “Boundary,” respectively (Model C), did not require any adjustment. The site use model assessing the effect of “Trees” was adjusted by adding the main effect of “River” (Model D).

**FIGURE 4:**
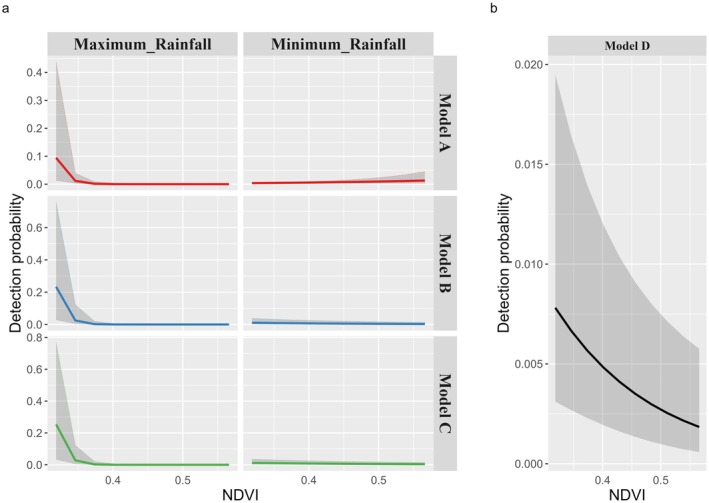
Estimates of the relationship between detection probability and (a) NDVI at maximum daily rainfall (mm) (“Maximum_Rainfall”) and NDVI at minimum daily rainfall (mm) (“Minimum_Rainfall”), across site use models A, B, and C; (b) NDVI in site use model D. The solid lines represent the predicted value of the detection probability and the shaded area the prediction interval at 95%, with colors associated to each site use model.

In three of the four site use models (Models A, B, C, Table 3), the top detection models included the same factors: the linear effect of both “NDVI” and “Rain” as well as the interaction term between those two covariates. The top detection in Model D only included the linear effect of “NDVI” (Table 3; complete ranking available as Data S1). All models showed no evidence of lack of fit nor overdispersion. Leopard detectability was negatively associated with “NDVI” in three of the four models (*β*_ModelA_ = 0.11 [−0.34 to 0.56], *β*_ModelB_ = −0.65 [−0.94 to −0.36], *β*_ModelC_ = −0.59 [−0.88 to −0.3], *β*_ModelD_ = −0.43 [−0.68 to −0.17]) and always negatively associated with “Rain” (*β*_ModelA_ = −0.53 [−0.96 to −0.1], *β*_ModelB_ = −0.54 [−0.97 to −0.11], *β*_ModelC_ = −0.54 [−0.97 to −0.11]). Additionally, the interaction term suggests leopard detectability was positively associated to an increase in rainfall in sites with the lowest vegetation productivity (Figure 4; all beta coefficients of Models A, B, C, and D are available as Data S1).

**TABLE 3:** Top models for site use and detection probability from a camera survey of African leopards ( *Panthera pardus pardus* ) using a Royle and Nichols (2003) model with temporally replicated detection–non‐detection data to estimate site‐specific heterogeneity detection probability (*p*), derived from site‐specific use of the species (*λ*). Models for site use probability (*λ*) were selected using causal inference, while models for detection probability (*p*) were selected using information criterion (AIC_C_). The goodness of fit of each model was assessed using the *mb.gof.test* function in the *AICcmodavg* package in R, which returns the *p*‐value (*p. value*) computed from the parametric bootstrap and the estimate of the overdispersion parameter (c.hat).

Model	*λ*	*p*	AIC_C_ *w*_*i*_	*p. value*	c.hat
A	NDVI + Trees + River	NDVI + Rain + NDVI × Rain	0.78	0.7	1.4 e‐12
B	River	NDVI + Rain + NDVI × Rain	0.98	0.4	1.5 e‐4
C	Boundary	NDVI + Rain + NDVI × Rain	0.98	1	1.6 e‐13
D	Trees + River	NDVI	0.54	0.7	1.9 e‐9

Models A, B, and C predicted that the probability of detecting leopards given presence at a site was maximum at lowest average NDVI values and at maximum rainfall (*p* = 0.09 [0.014–0.44] in model A; *p* = 0.23 [0.03–0.75] in model B; *p* = 0.25 [0.03–0.77] in model C). Model D predicted that the probability of detecting leopards given presence at a site was maximum at lowest vegetation productivity (*p* = 0.008 [0.003–0.02]; Figure 4).

The mean estimated probability of detecting a leopard during a single sampling occasion of one day was 0.5% ± 0.2% in Model A, 1.4% ± 0.3% in Models B and C, and 1% ± 0.3% in Model D. The estimated global detection probability was 80% ± 6.8% according to Model A, 98% ± 0.7% according to Model B, 98% ± 0.6% according to Model C, and 95% ± 2.3% according to Model D, based on 305 secondary occasions.

## 4 Discussion

Maximizing detection probability enhances the robustness of population and occupancy estimates for elusive species such as leopards (Kellner and Swihart 2014). We used occupancy modeling to provide inferences on the factors influencing the detectability of leopards in camera trap surveys in a semi‐arid wooded savannah ecosystem in Africa. We tested six different factors that we hypothesized may influence detection probability at the levels of encounter, trigger, and image following the framework of Hofmeester et al. (2019). We used a causal inference framework to provide four biologically robust site use models (Grace 2006) against which to test detection factors. The detection probability of all site use models was predicted by a reduced and consistent set of covariates, with only vegetation productivity and rainfall appearing as top factors associated with most of the site use models.

In this study, we found that vegetation productivity moderated by rainfall was the top predictor of leopard detectability. Our global detection probability were of similar magnitude to those reported by other studies on felids (99% ± 6e^−6^% in Strampelli et al. (2018); 85% ± 5% in Nichols et al. (2019) and 83% ± 6.2% in Stokeld et al. (2015)). Yet, the different scales of detection probability (“encounter,” “trigger,” and “image”) were “lumped” into a single parameter *p*, and our analytical framework (occupancy/RN models) did not allow to disentangle the influence of single covariates at a specific scale. However, at image scale, only 2.4% of the observations that might contain leopards could not be identified to species. Considering the low level of uncertainty at image scale, we further focused our interpretation of factors influencing detection probability at the “encounter” and “trigger” scales. Camera trapping studies that fail to account for habitat specificities (e.g., Rovero and Marshall 2009) implicitly assume that detection probability is constant across habitats. However, Hofmeester et al. (2017) demonstrated that differences between habitats can be large, with 20% decrease in transmission of infrared radiation in closed compared to open vegetation. In our study area, because high NDVI was correlated with dense habitat located in rough terrain (TRI), our results support the suggestion that increasing vegetation denseness lowered the probability of detection at trigger scale. Moreover, the negative association of rainfall with detectability could indicate that the presence of rain on the sensor of the camera may lower the sensitivity of the PIR sensor (Meek et al. 2014; Hofmeester et al. 2019) and result in decreasing trigger probability.

In our detection model, vegetation productivity, as measured with NDVI, was most often found to be interacting with rainfall as a moderating effect. We measured the average NDVI statically across the camera survey so that it could appear in both the site use and detection model components, while rainfall varied temporally and was restricted to the detection model only. The interaction between the temporally variable rainfall and the temporally static NDVI allowed the model to incorporate a seasonal change in vegetation. High rainfall in sites with moderate vegetation productivity positively influenced detection probability. This pattern may be reflecting a seasonal process in the biology of leopards and vegetation associated with encounter probability. Allen et al. (2020) observed that leopard distribution varied among the dry and wet seasons, and was primarily affected by interactions with other larger carnivores and vegetation cover, with leopards expanding habitat use in the wet season to areas with less cover. In the wet season, prey abundance is more evenly distributed throughout the landscape (Chaka et al. 2021; Kittle et al. 2017; Patterson et al. 2004), and leopards may use areas of moderate vegetation density more frequently when rainfall provides an additional element of secrecy during hunting (Jenny and Zuberbühler 2005). Conversely, high rainfall negatively influenced detection probability in sites with high vegetation productivity. We primarily attributed this pattern to quick vegetation growth in areas of high productivity, limiting the camera view shed and likely increasing false triggers. Despite regular vegetation clearing in front of the cameras, cameras were placed quite low to optimize leopard detection (at an average height of 44 cm off the ground) and were therefore prone to be obstructed again by quick vegetation growth within 2‐ or 3‐weeks' time. This is especially true for the April/May rainy season during which trap maintenance was operated on a bi‐monthly basis (instead of a monthly basis between as operated between August and December). Thus, the fast‐growing vegetation in rainy season might have negatively affected trigger probability. Yet, the scale at which we measured vegetation productivity, as the average NDVI within the 20 m radius buffer area around cameras, might not have provided precise enough information about vegetation triggers occurring at fine scale in front of the camera in wet season. It is therefore possible that the interaction between vegetation productivity and rainfall illustrates the relationship between vegetation density and detectability at encounter scale rather than at trigger scale for which we lacked precise measurement of vegetation blocking the view shed of the camera.

We found that the detection covariates trail, camera trap height, ambient temperature, and placement of the camera across from a tree were consistently excluded from top models predicting detection probability. As other large carnivores were present in our study area, we assumed that leopards would tend to avoid them by climbing trees (Stein et al. 2015; Balme et al. 2017). Thus, most of our cameras were facing suitable trees for leopards to rest, hoist prey (Stein et al. 2015; Balme et al. 2017) or scent mark (Smith 1977; Bothma and Le Riche 1984). Such camera placement was meant to optimize detectability by both channeling leopards into the detection zone (encounter probability) and increasing the time spent in front of the camera (trigger probability and image quality) (Hofmeester et al. 2019). However, the poor variability of this factor in our study design did not allow for assessing the influence of setting cameras across trees. This calls for further research to clarify the influence of such camera placement in leopard studies occurring in areas where competitors are also present. In our sample sites, high values of NDVI were associated with closed habitats, and the covariates “Trail” and “NDVI” were positively correlated, possibly because trails in dense vegetation are more likely to concentrate animal use (Kolowski and Forrester 2017). Leopard occupancy studies generally use trails or roads as detection covariates (e.g., McKaughan et al. 2024; Rich et al. 2016; Strampelli et al. 2018; Thapa et al. 2021), whereas we found that NDVI provided a better fit in our model. The presence of a trail in the detection zone of the camera can result in a positive influence on encounter probability, while the increase in both speed and directionality of a leopard on a trail may also negatively influence the trigger probability (Hofmeester et al. 2019). In contrast, the consistently negative influence of high vegetation density on detectability at all phases could possibly explain the stronger association of NDVI with detectability. It may also be possible that vegetation denseness is accounting for additional biological variation in detection that trails are not, especially when moderated with a seasonal covariate such as rainfall. However, our finding of vegetation density being an important detection component does not preclude the importance of placing cameras next to trails cutting through dense vegetation (Hunter et al. 2013). Leopards, in particular, use landscape features that facilitate movement when traveling and hunting (Hunter et al. 2013; Strampelli et al. 2018). Instead, we corroborate the findings of Kolowski and Forrester (2017) and recommend assessing vegetation density in the detection model in addition to landscape features like trails or roads.

Although camera height and ambient temperature have proved to influence detection probability in controlled experiments (McIntyre et al. 2020), in our study neither was determined to be important to leopard detection. As our camera grid was specifically set for leopards, camera heights were intended to be optimal at each site. We predicted that low ambient temperatures would have a positive influence on detections, as lower background surface temperatures tend to create a bigger infrared radiation contrast with animals characterized by higher surface temperatures (Swann et al. 2004). However, low ambient temperature can have antagonist effects on trigger probability. At low ambient temperature the PIR sensor better detects a difference in heat between leopards and the environment (Swann et al. 2004; McIntyre et al. 2020), which has a positive influence on trigger probability. Adversely, low temperature induces quick drainage of the batteries resulting in slower response time of the camera and lower PIR sensor sensitivity, which in turn negatively influences trigger probability (Cho et al. 2012). We modeled the non‐linear effects of temperature using a quadratic term in the detection model, but it did not sufficiently increase the fit for inclusion. Mean daily ambient temperatures were measured as the average temperature over our whole study area rather than site specific, and given the equatorial setting of our study, limited variation may have reduced the need for the inclusion of this as an important detection factor.

While optimizing detection probability improves estimates of population parameters, the cost effectiveness of the study designs is also an essential consideration for long‐term monitoring of elusive species. Thus, emphasis should be drawn to detection factors for which adjustments involve minimal cost, as many practitioners cope with limited financial resources, especially in the Global South (Bruner et al. 2004). Most biases impacting the probability of obtaining a good‐quality image can relatively easily be accounted for by standardizing camera trap model and camera trap set‐up protocols (Hofmeester et al. 2019; McIntyre et al. 2020). In contrast, factors influencing the encounter probability and the trigger probability are the most important issues to consider (Hofmeester et al. 2019, 2021). Among those, the placement of camera traps involves minimal cost when adequately accounted for in the early stages of the grid design. We found that setting cameras in microhabitat of moderate productivity improved leopard detectability in the wet season and required only simple and easily obtainable measures for detection covariates. Our results can inform the design of camera trap studies occurring in semi‐arid bushland ecosystems to improve estimates of leopard population and occupancy.

## Author Contributions

**Beatrice Chataigner:** conceptualization (equal), data curation (equal), formal analysis (lead), investigation (equal), visualization (equal), writing – original draft (lead), writing – review and editing (lead). **Nicholas W. Pilfold:** conceptualization (equal), data curation (equal), funding acquisition (lead), visualization (equal), writing – original draft (supporting), writing – review and editing (equal). **Laiyon Lenguya:** data curation (equal), investigation (equal). **Aurélien G. Besnard:** conceptualization (equal), writing – review and editing (equal). **Olivier Gimenez:** conceptualization (equal), writing – original draft (supporting), writing – review and editing (equal).

## Conflicts of Interest

The authors declare no conflicts of interest.

## Supporting information


Data S1.

## Data Availability

The African leopard is a species with high conservation stakes considered Vulnerable to extinction by the International Union for the Conservation of Nature (Stein et al. 2025). The hunting of leopards is illegal in Kenya and this species is listed as endangered in the Kenyan Wildlife Conservation and Management Act. In addition, leopards in our study area include melanistic individuals (Pilfold et al. 2019a), one of the few known areas in Africa where they are documented to reside (da Silva et al. 2017). Because interactions with human activities are problematic and lead to poaching, retaliation, and other anthropogenic pressures, providing accurate information on leopards' locations can be detrimental to the conservation status of the species, including heightening the poaching risk to individuals that may be perceived to be of high economic value for trade. For these reasons, research data are not shared.
