# Supplementary material for: Unusual Suspects: Detection Probability Covaries With Vegetation Productivity and Rainfall for Camera Survey of African Leopards ( Panthera pardus pardus )
Source: Ecol Evol. 2025 May 14;15(5):e71346. doi: 10.1002/ece3.71346 (PMC12077962; doi:10.1002/ece3.71346)
Supplement: Supplementary file 1 — Data S1. [file ECE3-15-e71346-s001.docx]

**Supporting Information**

Unusual suspects: Detection probability covaries with vegetation productivity and rainfall for camera survey of African leopards (*Panthera pardus pardus*)

Beatrice Chataigner^1*^, Nicholas W. Pilfold^2,^ Laiyon Lenguya^3^, Aurélien G. Besnard^1^, Olivier Gimenez^4^

**Affiliations**

^1^ CEFE, Univ Montpellier, CNRS, EPHE-PSL University, IRD, Montpellier, France

^2^ San Diego Zoo Wildlife Alliance, San Diego, United States

^3^ Loisaba Conservancy, Nanyuki, Kenya

^4^ CEFE, Univ Montpellier, CNRS, EPHE, IRD, Montpellier, France

**Corresponding author**

Beatrice Chataigner, [beatricechataigner@gmail.com](mailto:beatricechataigner@gmail.com), [orcid.org/0009-0008-7755-6775](https://orcid.org/0009-0008-7755-6775?lang=en), CEFE, Univ Montpellier, CNRS, EPHE-PSL University, IRD, Montpellier, France.

**Table S1** AICc ranking of the detection models associated to the site use model meant to assess the effect of “NDVI” conditioned by “Trees” and “River” as a result of the backdoor criterion analysis. Covariate key: Average vegetation productivity at site (NDVI); Distance to nearest river (River); Mean diameter at breast height of trees present at site (Trees); Diameter at breast height of tree within camera viewshed (CT_Tree); Presence of a trail at camera site (Trail); Mean daily rainfall (Rain); Mean daily temperature (Temp); Height of camera off the ground (CT_Height); (null) signifies constant parameter. Delta_AICc = Difference between model AICc and that of model with the lowest AICc; AICcWt = relative model weight; K = number of parameters in the model; LL = twice the negative likelihood.

| **id** | **Models** | **Delta_AICc** | **AICcWt** | **K** | **LL** |
| --- | --- | --- | --- | --- | --- |
| 1 | p(NDVI * Rain) lambda(NDVI + Trees + River) | 0.00 | 0.78 | 8 | -642.16 |
| 2 | p(null) lambda(NDVI + Trees + River) | 4.19 | 0.10 | 5 | -650.39 |
| 3 | p(Trail) lambda(NDVI + Trees + River) | 6.90 | 0.02 | 6 | -649.94 |
| 4 | p(NDVI) lambda(NDVI + Trees + River) | 6.95 | 0.02 | 6 | -649.96 |
| 5 | p(CT_Height) lambda(NDVI + Trees + River) | 7.50 | 0.02 | 6 | -650.24 |
| 6 | p(Rain) lambda(NDVI + Trees + River) | 7.52 | 0.02 | 6 | -650.25 |
| 7 | p(Temp * Temp) lambda(NDVI + Trees + River) | 7.75 | 0.02 | 6 | -650.36 |
| 8 | p(CT_Tree) lambda(NDVI + Trees + River) | 7.79 | 0.02 | 6 | -650.39 |
| 9 | p(NDVI + Rain) lambda(NDVI + Trees + River) | 10.74 | 0.00 | 7 | -649.83 |
| 10 | p(Temp + Rain) lambda(NDVI + Trees + River) | 11.40 | 0.00 | 7 | -650.16 |
| 11 | p(CT_Tree + Trail + CT_Height + NDVI + Rain + Temp) lambda(NDVI + Trees + River) | 29.34 | 0.00 | 11 | -647.63 |

**Table S2** β-coefficients (Estimates) and associated standard error estimates (SE) of the top model (**model A**) coming out of the AICc ranking of the detection models associated to the site use model meant to assess the effect of vegetation productivity (NDVI), conditioned by the mean diameter at breast height of trees present at site (Trees) and by the distance to nearest river (River) as a result of the backdoor criterion analysis.

| **p(Rain + NDVI + Rain * NDVI) lambda(NDVI + River + Trees)** | **Estimate** | **SE** | **z** | **P(>\|z\|)** |
| --- | --- | --- | --- | --- |
| (Intercept_p) | -5.26 | 0.27 | -19.16 | 0 |
| Rain | -0.53 | 0.22 | -2.40 | **0.02** |
| NDVI | 0.11 | 0.23 | 0.49 | 0.62 |
| Rain:NDVI | -0.71 | 0.24 | -2.97 | **0** |
| (Intercept_lambda) | 1.89 | 0.27 | 7.09 | 0 |
| NDVI. | -0.71 | 0.24 | -2.97 | **0** |
| River | -0.02 | 0.12 | -0.15 | 0.88 |
| Trees | 0.26 | 0.14 | 1.89 | 0.06 |

**Table S3** AICc ranking of the detection models associated to the site use model meant to assess the effect of “River”. Covariate key: Average vegetation productivity at site (NDVI); Distance to nearest river (River); Diameter at breast height of tree within camera viewshed (CT_Tree); Presence of a trail at camera site (Trail); Mean daily rainfall (Rain); Mean daily temperature (Temp); Height of camera off the ground (CT_Height); (null) signifies constant parameter. Delta_AICc = Difference between model AICc and that of model with the lowest AICc; AICcWt = relative model weight; K = number of parameters in the model; LL = twice the negative likelihood.

| **id** | **Models** | **Delta_AICc** | **AICcWt** | **K** | **LL** |
| --- | --- | --- | --- | --- | --- |
| 1 | p(NDVI * Rain) lambda(River) | 0.00 | 0.99 | 6 | -647.79 |
| 2 | p(NDVI) lambda(River) | 9.02 | 0.01 | 4 | -655.72 |
| 3 | p(NDVI + Rain) lambda(River) | 11.97 | 0.00 | 5 | -655.58 |
| 4 | p(null) lambda(River) | 15.71 | 0.00 | 3 | -660.51 |
| 5 | p(Trail) lambda(River) | 17.55 | 0.00 | 4 | -659.98 |
| 6 | p(Rain) lambda(River) | 18.37 | 0.00 | 4 | -660.39 |
| 7 | p(Temp * Temp) lambda(River) | 18.48 | 0.00 | 4 | -660.45 |
| 8 | p(CT_Height) lambda(River) | 18.54 | 0.00 | 4 | -660.48 |
| 9 | p(CT_Tree) lambda(River) | 18.61 | 0.00 | 4 | -660.51 |
| 10 | p(Temp + Rain) lambda(River) | 21.27 | 0.00 | 5 | -660.23 |
| 11 | p(CT_Tree + Trail + CT_Height + NDVI + Rain + Temp) lambda(River) | 27.55 | 0.00 | 9 | -654.61 |

**Table S4** β-coefficients (Estimates) and associated standard error estimates (SE) of the top model (**model B**) coming out of the AICc ranking of the detection models associated to the site use model meant to assess the effect of the distance to nearest river (River).

| **p(Rain + NDVI + Rain * NDVI) lambda(River)** | **Estimate** | **SE** | **z** | **P(>\|z\|)** |
| --- | --- | --- | --- | --- |
| (Intercept_p) | -5.20 | 0.68 | -7.64 | 0 |
| Rain | -0.54 | 0.22 | -2.41 | **0.02** |
| NDVI | -0.65 | 0.15 | -4.21 | **0** |
| Rain:NDVI | -0.73 | 0.24 | -2.97 | **0** |
| (Intercept_lambda) | 1.86 | 0.67 | 2.78 | 0.01 |
| River | -0.11 | 0.12 | -0.92 | 0.36 |

**Table S5** AICc ranking of the detection models associated to the site use model meant to assess the effect of “Boundary”. Covariate key: Distance to conservancy border (Boundary); Average vegetation productivity at site (NDVI); Diameter at breast height of tree within camera viewshed (CT_Tree); Presence of a trail at camera site (Trail); Mean daily rainfall (Rain); Mean daily temperature (Temp); Height of camera off the ground (CT_Height); (null) signifies constant parameter. Delta_AICc = Difference between model AICc and that of model with the lowest AICc; AICcWt = relative model weight; K = number of parameters in the model; LL = twice the negative likelihood.

| **id** | **Models** | **Delta_AICc** | **AICcWt** | **K** | **LL** |
| --- | --- | --- | --- | --- | --- |
| 1 | p(NDVI * Rain) lambda(Boundary) | 0.00 | 0.99 | 6 | -648.19 |
| 2 | p(NDVI) lambda(Boundary) | 8.97 | 0.01 | 4 | -656.09 |
| 3 | p(NDVI + Rain) lambda(Boundary) | 11.93 | 0.00 | 5 | -655.96 |
| 4 | p(null) lambda(Boundary) | 15.07 | 0.00 | 3 | -660.59 |
| 5 | p(Trail) lambda(Boundary) | 17.04 | 0.00 | 4 | -660.13 |
| 6 | p(Rain) lambda(Boundary) | 17.73 | 0.00 | 4 | -660.47 |
| 7 | p(Temp * Temp) lambda(Boundary) | 17.85 | 0.00 | 4 | -660.53 |
| 8 | p(CT_Height) lambda(Boundary) | 17.89 | 0.00 | 4 | -660.55 |
| 9 | p(CT_Tree) lambda(Boundary) | 17.96 | 0.00 | 4 | -660.58 |
| 10 | p(Temp + Rain) lambda(Boundary) | 20.62 | 0.00 | 5 | -660.30 |
| 11 | p(CT_Tree + Trail + CT_Height + NDVI + Rain + Temp) lambda(Boundary) | 27.17 | 0.00 | 9 | -654.82 |

**Table S6** β-coefficients (Estimates) and associated standard error estimates (SE) of the top model (**model C**) coming out of the AICc ranking of the detection models associated to the site use model meant to assess the effect of the distance to conservancy border (Boundary).

| **p(Rain + NDVI + Rain * NDVI) lambda(Boundary)** | **Estimate** | **SE** | **z** | **P(>\|z\|)** |
| --- | --- | --- | --- | --- |
| (Intercept_p) | -5.04 | 0.60 | -8.45 | 0 |
| Rain | -0.54 | 0.22 | -2.40 | **0.02** |
| NDVI | -0.59 | 0.15 | -3.97 | **0** |
| Rain:NDVI | -0.73 | 0.25 | -2.95 | **0** |
| (Intercept_lambda) | 1.69 | 0.58 | 2.91 | 0 |
| Boundary | -0.03 | 0.12 | -0.25 | 0.8 |

**Table S7** AICc ranking of the detection models associated to the site use model meant to assess the effect of “Trees” conditioned by “River” as a result of the backdooor criterion analysis. Covariate key: Mean diameter at breast height of trees present at site (Trees); Average vegetation productivity at site (NDVI); Distance to nearest river (River); Diameter at breast height of tree within camera viewshed (CT_Tree); Presence of a trail at camera site (Trail); Mean daily rainfall (Rain); Mean daily temperature (Temp); Height of camera off the ground (CT_Height); (null) signifies constant parameter. Delta_AICc = Difference between model AICc and that of model with the lowest AICc; AICcWt = relative model weight; K = number of parameters in the model; LL = twice the negative likelihood.

| **id** | **Models** | **Delta_AICc** | **AICcWt** | **K** | **LL** |
| --- | --- | --- | --- | --- | --- |
| 1 | p(NDVI) lambda(River + Trees) | 0.00 | 0.64 | 5 | -652.15 |
| 2 | p(NDVI + Rain) lambda(River + Trees) | 3.33 | 0.12 | 6 | -652.01 |
| 3 | p(Trail) lambda(River + Trees) | 3.53 | 0.11 | 5 | -653.91 |
| 4 | p(null) lambda(River + Trees) | 4.79 | 0.06 | 4 | -656.15 |
| 5 | p(CT_Tree) lambda(River + Trees) | 6.63 | 0.02 | 5 | -655.46 |
| 6 | p(Rain) lambda(River + Trees) | 7.77 | 0.01 | 5 | -656.03 |
| 7 | p(Temp * Temp) lambda(River + Trees) | 7.87 | 0.01 | 5 | -656.08 |
| 8 | p(CT_Height) lambda(River + Trees) | 8.01 | 0.01 | 5 | -656.15 |
| 9 | p(Temp + Rain) lambda(River + Trees) | 11.03 | 0.00 | 6 | -655.85 |
| 10 | p(CT_Tree + Trail + CT_Height + NDVI + Rain + Temp) lambda(River + Trees) | 17.17 | 0.00 | 10 | -648.93 |
| 11 | p(NDVI * Rain) lambda(River + Trees) | 671.72 | 0.00 | 7 | -984.17 |

**Table S8** β-coefficients (Estimates) and associated standard error estimates (SE) of the top model (**model D**) coming out of the AICc ranking of the detection models associated to the site use model meant to assess the effect of the mean diameter at breast height of trees present at site (Trees), conditioned by the distance to nearest river (River) as a result of the backdooor criterion analysis.

.

| **p(NDVI) lambda(River + Trees)** | **Estimate** | **SE** | **z** | **P(>\|z\|)** |
| --- | --- | --- | --- | --- |
| (Intercept_p) | -5.39 | 0.47 | -11.47 | 0 |
| NDVI | -0.43 | 0.13 | -3.25 | **0** |
| (Intercept_lambda) | 2.10 | 0.47 | 4.49 | 0 |
| River | 0.03 | 0.12 | 0.24 | 0.81 |
| Trees | 0.35 | 0.13 | 2.73 | **0.01** |
